# Intra-articular osteoid osteoma as a differential diagnosis of diffuse mono-articular joint pain

**DOI:** 10.1186/s12891-016-1313-3

**Published:** 2016-11-04

**Authors:** Tim Rolvien, Jozef Zustin, Haider Mussawy, Tobias Schmidt, Pia Pogoda, Peter Ueblacker

**Affiliations:** 1 Department of Osteology and Biomechanics, University Medical Centre Hamburg-Eppendorf, Lottestr. 59, Hamburg, 22529 Germany; 2 MW Centre for Orthopaedics and Sports Medicine, Munich, Germany

**Keywords:** Intra-articular osteoid osteoma, Differential diagnosis, Joint pain, Bone tumour, Imaging

## Abstract

**Background:**

The aim of this retrospective study was to investigate the frequency of intra-articular osteoid osteoma (iaOO) in a large study cohort and to demonstrate its clinical relevance as an important differential diagnosis of non-specific mono-articular joint pain.

**Methods:**

We searched the registry for bone tumours of the University Medical Centre Hamburg-Eppendorf for osteoid osteomas in the last 42 years. Herein, we present three selected iaOO which were detected in the three major weight-bearing joints. Computed tomography (CT) or magnetic resonance imaging (MRI) scans were performed for initial diagnosis.

**Results:**

Out of a total of 367 osteoid osteomas, 19 (5.2 %) tumours were localized intra-articularly. In all three presented tumours, a history of severe mono-articular pain was reported; however, the mean time to correct diagnosis was delayed to 20.7 months. Clearly, the nidus seen in CT and MRI images in combination with inconsistent salicylate-responsive nocturnal pain led to the diagnosis of iaOO.

**Conclusions:**

Rarely, osteoid osteoma can occur in an intra-articular location. In cases of diffuse mono-articular pain, iaOO should be considered both in large and smaller joints to avoid delays in diagnosis and therapy of this benign bone tumour.

**Electronic supplementary material:**

The online version of this article (doi:10.1186/s12891-016-1313-3) contains supplementary material, which is available to authorized users.

## Background

Osteoid osteoma (OO) was first described as a specific entity by Jaffe in 1935 [1] and is the third most common benign lesion of bone. It is an osteoblastic tumour that produces unmineralized bone called osteoid and is accompanied by a central area of calcification called nidus, that is usually seen in computed tomography (CT) [2]. OO may be localized cortically, trabecularly or subperiosteally/subchondrally [3]. Adolescents and young adults are mostly affected and present with nocturnal pain that responds to nonsteroidal anti-inflammatory drugs (NSAIDs), especially salicylates, in the majority of cases.

Localizations within the skeleton may vary, although the diaphysis in long bones of the lower extremity is most commonly affected. Intra- and juxta-articular localizations have been described, but only account for around 10 % of OO [4–9]. Especially in intra-articular osteoid osteoma (iaOO), diagnosis might be prolonged due to diffuse joint pain, which is initially assigned to more common differential diagnoses, including inflammatory joint diseases or osteochondritis dissecans [10]. In fact, when compared with extra-articular localization, diagnosis has been shown to be significantly delayed (26.6 vs. 8.5 months) [11].

Besides making the correct diagnosis, the challenge is that various therapeutical options including arthroscopic removal and radiofrequency ablation (RFA) are available [9, 12–14]. Correct localization of the tumour on one hand but protection of joint cartilage on the other are the primary objectives. In the present study, we aim to investigate and review the frequency of iaOO in our large registry of bone tumours and illustrate its differential diagnosis among three particular cases.

## Methods

This retrospective study searched the registry for bone tumours at the University Medical Centre Hamburg-Eppendorf for all detected osteoid osteomas from 1974 to 2016. Age, sex, radiological investigations, tumour location, histopathological features including type and dignity of the tumour and diagnosis were documented. The registry collected both slides and tissue blocks (both paraffin and methyl-methacrylate) from tumour biopsies and resection specimens as well. All clinically assumed OO that could not be confirmed histologically were excluded. Therefore, 367 cases with histopathologically diagnosed OO were included in this study. Age and gender distribution, as well as the frequency of iaOO among all OO was evaluated with Microsoft Excel software.

Haematoxylin and eosin (paraffin blocks) and toluidine blue staining methods (methyl-methacrylate embedded blocks) were performed for the purpose of the present study. Microscopy was carried out using a Zeiss microscope (Axiophot, Carl Zeiss, Jena, Germany) and photographs taken with AxioVision Rel.4.8 (Carl Zeiss, Jena, Germany). Furthermore, three of the most recent cases of iaOO of the lower extremity were selected and are presented here. These cases were all seen and clinically diagnosed at our institution.

## Results & case series

**Fig. 1:**
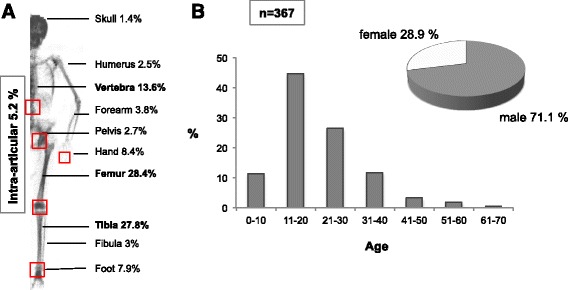
Distribution of OO in 367 cases. **a** Most OO were localized within the diaphysis of the femur followed by tibia and vertebra. Intra-articular localization was only found in 19 (5.2 %) of the cases. Red boxes highlight the joints in which iaOO were found. Whole body scan obtained by dual-energy X-ray absorptiometry (DXA). **b** Age and gender distribution

367 osteoid osteomas were found, while most cases were located in the femur (28.4 %), followed by localization in the tibia (27.8 %) and in the vertebra (13.6 %, Fig. 1a). The occurrence of OO was most frequent in the second decade of life and then continuously declined with age (Fig. 1b). From 367 detected OO, 19 (5.2 %) were localized intra-articularly. This involved localization in the proximal (*n* = 3) and distal (*n* = 1) tibia, proximal (*n* = 2) and distal femur (*n* = 1), spine (*n* = 2), talus (*n* = 4), calcaneus (*n* = 2) and hand (*n* = 4), respectively (Table 1). All iaOO occurred between the 2^nd^ and 4^th^ life decade with a male:female ratio of 2:1.

**Table 1:** Absolute numbers of osteoid osteoma for each skeletal region

Joint location	n (total)	n (intra-articular)
Femur	105	3
Tibia	101	4
Hand	31	4
Foot	29	6
Lumbar spine	21	1
Thoracic spine	15	1
Cervical spine	14	-
Fibula	11	-
Pelvis	10	-
Humerus	9	-
Radius	8	-
Ulna	6	-
Skull	5	-
Ribs	1	-
Clavicle	1	-
Total	367	19

### Case 1: 26-year-old male with osteoid osteoma of the ankle joint (talus)

A 26-year-old male presented with persistent and non-specific medial ankle pain in the left ankle that he had had for 32 months in total. A minor trauma while playing basketball was reported, though the pain persisted over the following months. A pain diary was started and revealed pain levels of 5–7 on the Visual Analogue Scale (VAS) at rest, occurring predominantly at night. No typical response to salicylates was reported in the beginning. Physical examination showed no signs of ankle instability. Arthroscopy was performed due to suspected synovitis but histology revealed that this was not the case. Extended laboratory tests did not point to any rheumatological diseases. Furthermore, multiple intra-articular injections were unsuccessful in terms of pain reduction. The lesion seen in CT and repeated MRI scans was eventually interpreted as an intra-articular, subchondral osteoid osteoma (3.1 × 2.7 mm) in the anterior process of the talus (Fig. 1a), although in the initial MRI there was no suspicion of iaOO. The patient was advised to undergo arthroscopic resection of the tumour.

### Case 2: 26-year-old male with osteoid osteoma of the knee joint (proximal tibia)

Another 26-year-old patient presented at our clinic with non-specific anterior knee pain after having consulted different orthopaedic surgeons that had suspected meniscus injury, bone marrow oedema and anterior cruciate ligament instability. The patient had worsening pain in the lateral knee, but no trauma was reported. Clinical examination revealed no signs of knee instability. Partial meniscectomy had been performed due to a submeniscal bone impression seen in MRI images and a lateral meniscus tear seen in arthroscopy. However, this intervention did not achieve any improvement of symptoms.

**Fig. 2:**
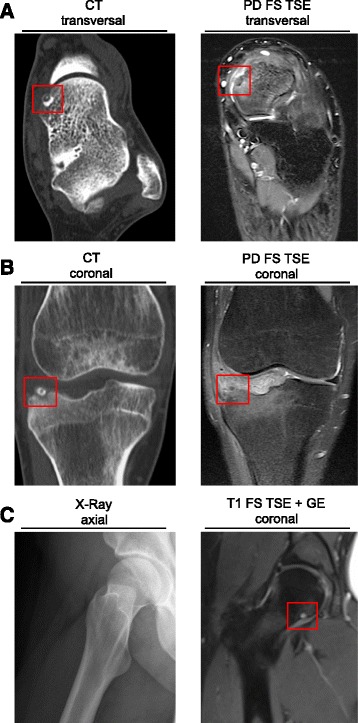
Imaging of iaOO. **a** *Case 1:* Coronal reformat of a CT scan showing a subchondral OO with central calcification (nidus) in the talar neck adjacent to the distal tibia (*red box*). In proton density (PD)-weighted fat-suppressed (FS) turbo spin echo (TSE) images, the nidus was not as apparent as in CT scans. **b** *Case 2:* iaOO in the lateral tibia plateau, coronal CT reconstruction (*red box*). In PD FS TSE MRI, the nidus is only pictured as ill-defined signal attenuation in the subchondral bone of the lateral tibia. **c** *Case 3:* X-Ray does not show any pathological findings. Gadolinium-enhanced (GE) MR imaging (FS, T1-weighted, TSE) shows circular enhancement of the subchondral bone of the femoral head (red box). For more MRI sequences see Additional file 1: Figure S1

**Fig. 3:**
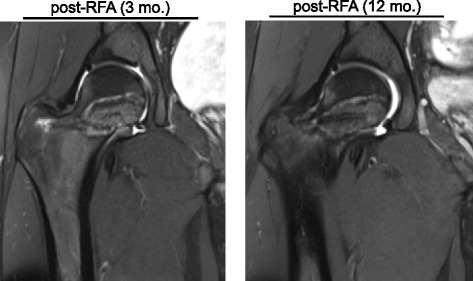
Femoral neck osteonecrosis after RFA. Proton density (PD)-weighted fat-suppressed (FS) turbo spin echo (TSE) MRI images 3 and 12 months after the intervention. There is a hyperintense signal indicating an excessive necrosis zone around the insertion channel of the ablation probe

Based on the patient’s information about resolving nocturnal pain following aspirin medication, we performed a CT scan that showed the typical nidus at the lateral tibia plateau, which was not as obvious in MRI scans (Fig. 2b). Time until diagnosis was 2 years in total. The arthroscopic resection was successful and led to a full recovery of the patient’s symptoms. Histology of the tumour biopsy confirmed the diagnosis (Fig. 3). At 3 month follow-up the patient was still free of pain and had a full range of motion.

### Case 3: 22-year-old female with osteoid osteoma in the hip joint (femoral head)

This 22-year old female patient was referred to our outpatient clinic. The patient was an enthusiastic tennis player and had had diffuse pain in the right hip joint for more than 6 months. Nocturnal pain was not the leading symptom; rather, joint tenderness and a restricted range of motion was reported. External leading differential diagnoses in the beginning were synovitis and coxa saltans. Conventional radiographs were inconspicuous (Fig. 2c). Due to a risk of high radiation exposure, the patient did not wish a CT scan to be performed. Instead, a dynamic gadolinium-enhanced MRI scan was carried out. Coronal reconstruction of a fat-suppressed (FS), T1-weighted, turbo spin echo (TSE) MRI showed a 2.8 × 3.5 mm nidus in the right femoral head (Fig. 2c). T1-weighted images without contrast enhancement revealed a hypointense signal, while in T2-weighted sequences the signal was equally hyperintense (Additional file 1: Figure S1). The patient was advised to undergo RF ablation of the tumour.

RF ablation of the OO was performed in order to induce osteonecrosis of the nidus. However, it led to subsequent osteonecrosis of almost the complete femoral neck, which was diagnosed in post-interventional MRI images 3 months after the intervention. In proton density (PD)-weighted images altered signal intensity around the insertion channel of the RF ablation probe pointed to an accompanying marrow oedema. In a 1 year follow-up MRI there was little joint effusion and a constant necrosis zone (Fig. 3).

Full osteologic assessment including laboratory analyses and dual-energy X-ray absorptiometry (DXA; Lunar iDXA, GE Healthcare, Madison, WI, USA) was performed and did not point to any primary or secondary bone diseases which could have provoked the osteonecrosis. In fact, DXA revealed a normal bone mineral density (BMD). We advised the patient to avoid uncontrolled movements in order not to risk further necrosis of the femoral head, although she was by then nearly symptom-free at rest. Imbalanced vitamin D and calcium homeostasis in terms of a moderate vitamin D insufficiency (25-OH-D3 20 μg/l) was controlled by daily supplementation with 2,000 IE vitamin D.

## Discussion

Osteoid osteoma is a bone-forming tumour with typical localization in the diaphysis of long bones; however, it can be particularly challenging when localized intra-articularly [11, 15]. We investigated the frequency of iaOO in a large tumour registry and present three cases of iaOO of the lower extremity, all of them initially clinically misinterpreted. While OO is reported to be the third most common benign bone tumour in the literature and iaOO make up around 10–12 % of all OO [8, 9, 11], we identified only 5.2 % iaOO in our large collection of OO. This means that the overall percentage of iaOO might be lower than previously reported; however, our data was collected from a histological database, where clinically diagnosed OO and iaOO without histologically confirmed diagnosis were not included. In fact, the differentiation between intra- and juxta-articular localization of OO is sometimes not clear, although the diagnostic criterion for iaOO is its localization within the joint capsule. OO are usually cortical bone tumours, while the degree of joint involvement (i.e. entirely cortical, periosteal, subchondral) might not easily be estimated.

Clinically, the iaOO shown here did not consistently show nocturnal pain. Furthermore, in all three cases plain radiographies were inconspicuous. Other differential diagnoses including synovitis, rheumatological diseases and meniscus or ligament tearing were initially taken into account before suspecting OO. These suspected diagnoses were often the reason for initially carrying out an MRI and subsequent misdiagnosis [16]. Furthermore, early stages of the tumour, non-specific joint pain and a lack of experience may lead to a delayed diagnosis in general. In fact, MRI alone did not clearly allow diagnosis of OO in *Cases 1* and *2*. Considering all diagnostic options, CT imaging remained the most specific technique to detect iaOO [2, 7, 17], especially as a tumour size of under 3 mm may lead to misinterpretation of MRI images as described in *Case 2* [17]. Nevertheless, if MRI is performed, gadolinium-enhanced imaging was shown to be useful in terms of nidus conspicuity in iaOO [18, 19] which may have been the reason for a relatively early diagnosis in *Case 3*. Radionuclide imaging, which was reported to be a sensitive diagnostic modality [20], was not performed in our institution, especially since CT or MRI eventually allowed the correct diagnosis.

**Fig. 4:**
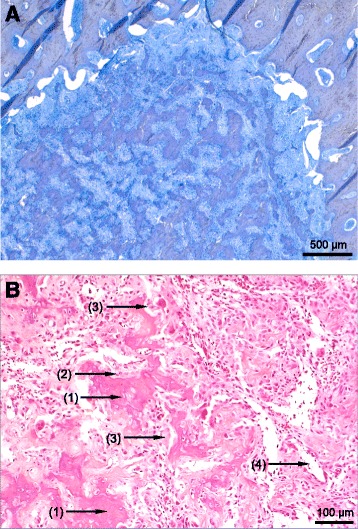
Osteoid osteoma, microscopic findings. **a** Overview. The cellular tumour (mid and lower left) shows irregular bone trabeculae partly consisting of osteoid and embedded in fibrous stroma. Cortical bone (upper left and upper and lower right) appears sclerotic. **b** Detail. The tumour tissue consists of woven bone trabeculae (1) covered by enlarged osteoblasts (2) and scattered multinucleated osteoclasts (3). The fibrous stroma contains dilated blood vessels (4). There is no evidence of tumour necrosis or cellular atypia. **a** Undecalcified preparation, staining method: toluidine blue, original magnification: 25×; **b** EDTA decalcification, staining method: haematoxylin and eosin, original magnification: 200 ×

There are several possibilities for therapy of the tumour: a main advantage of the surgical removal of iaOO is the possibility of histopathological diagnosis. The histology shows irregular immature woven bone trabeculae covered by activated osteoblasts and embedded in vascular fibrous stroma with scattered osteoclasts (Fig. 4a and b). In minimal invasive techniques like RF ablation (RFA), the nidus of the OO is usually heated to 90 °C for 4–9 min to achieve thermal coagulation necrosis which makes it impossible to perform histological examination [21–23]. Furthermore, the area of osteonecrosis might not be easy to control as RFA has been associated with occurring damage of the articular cartilage in the acetabulum [24].

We here present the first case in the literature where RFA led to excessive osteonecrosis of the femoral neck. Most likely this was because there was either too much heat administered, an inappropriate device was used or the total ablation time was too long. We conclude that RFA is only safe when the induced osteonecrosis can be controlled. Especially when localized intra- or juxta-articularly, we highly suggest considering RFA with care, as osteonecrosis was a direct consequence of this procedure. In our case, it has to be expected that the necrosis zone will only revitalize very slowly, if at all. The use of alternative methods including tissue impedance as a parameter of osteonecrosis [25] or magnetic resonance-guided focused ultrasound [26] might be promising and gentle alternatives. Nonetheless, further investigation including large multi-center studies is needed to compare outcomes between these new methods and RFA. Although arthroscopic surgery and RFA, or even a combination of both, have been shown to be equivalently successful in the therapy of iaOO [27–29], we here outline potential risks of RFA.

## Conclusions

In conclusion, we found 19 iaOO within a total of 367 OO (5.2 %) in our large registry of bone tumours. Furthermore, we demonstrate three cases of iaOO, which all atypically presented with non-specific joint pain and subsequent initial incorrect diagnosis. Based on our data, we recommend considering iaOO especially in young patients presenting with persistent joint pain of both small and large joints. Furthermore, we suggest arthroscopy with subsequent removal of the tumour and histopathological work-up of the lesion.

## Additional file

Additional file 1: Figure S1. The osteoid osteoma in *Case 3* had a hypointense signal in T1-weighted sequences (*left panel*), while it showed strong contrast enhancement in T1-weighted gadolinium-enhanced (GE) images (*middle panel*). In T2-weighted images, the signal was equally hyperintense (*right panel*). (PDF 3517 kb)

## Abbreviations

BMD: Bone mineral density
CT: Computed tomography
DXA: Dual-energy X-ray absorptiometry
iaOO: Intra-articular osteoid osteoma
MRI: Magnetic resonance imaging
OO: Osteoid osteoma
RFA: Radiofrequency ablation
