# Supplementary figures and images for: Intra-articular osteoid osteoma as a differential diagnosis of diffuse mono-articular joint pain
Source: BMC Musculoskelet Disord. 2016 Nov 4;17:455. doi: 10.1186/s12891-016-1313-3 (PMC5096000; doi:10.1186/s12891-016-1313-3)

Supplemental Figure

T1 TSE coronal

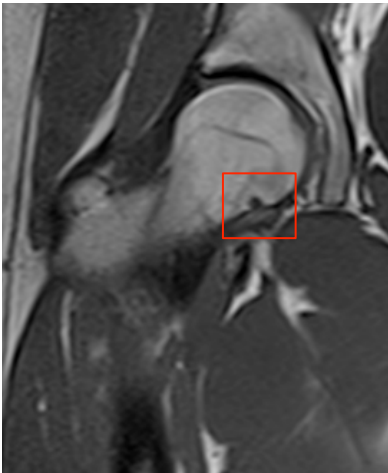

T1 FS TSE + GE  
coronal

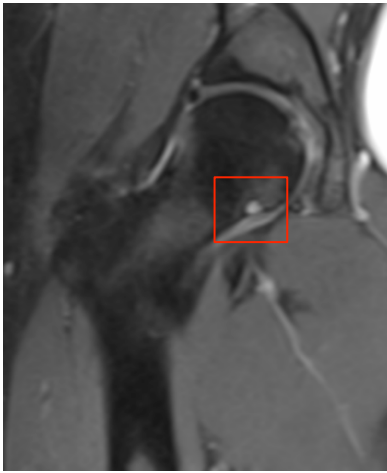

T2 FS TSE coronal

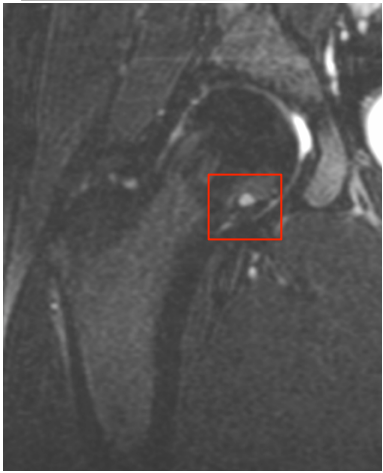

Supplement: Additional file 1: Figure S1. — The osteoid osteoma in Case 3 had a hypointense signal in T1-weighted sequences (left panel), while it showed strong contrast enhancement in T1-weighted gadolinium-enhanced (GE) images (middle panel). In T2-weighted images, the signal was equally hyperintense (right panel). (PDF 3517 kb) [file 12891_2016_1313_MOESM1_ESM.pdf]
